# Assessment of Excess Mortality Associated With Drug Overdose in Ohio From 2009 to 2018

**DOI:** 10.1001/jamanetworkopen.2020.2183

**Published:** 2020-04-07

**Authors:** O. Trent Hall, Orman E. Hall, Andrew Kolodny, Julie Teater, Ryan P. McGrath

**Affiliations:** 1 Department of Psychiatry and Behavioral Health, Ohio State University Wexner Medical Center, Columbus; 2 College of Health Sciences and Professions, Ohio University, Athens; 3 Opioid Policy Research Collaborative, Heller School for Social Policy and Management, Brandeis University, Waltham, Massachusetts; 4 Department of Health, Nutrition, and Exercise Sciences, North Dakota State University, Fargo

## Abstract

This cross-sectional study examines excess mortality, measured as years of life lost, associated with unintentional drug overdose in Ohio from 2009 to 2018.

## Introduction

Despite sustained efforts to reduce drug overdose mortality, drug overdoses continue to be an issue of significant concern in the US.^1^ The state of Ohio has the second highest incidence of fatal drug overdose; therefore, Ohio remains an important bellwether of this evolving national issue.^2^ A 2019 study^3^ reported that more than 500 000 years of life were lost to opioid overdose in Ohio from 2010 to 2016 and that opioid overdose had a measurable effect on life span in the state. However, more work is needed to contextualize these opioid deaths within the broader context of drug misuse and overdose and in relation to other leading causes of mortality. This study builds on our 2019 report^3^ of overdose mortality burden in Ohio to provide such context.^4^

## Methods

This cross-sectional study included data from death records obtained from the Ohio Department of Health. The institutional review board of Ohio University determined this study to be nonregulated, and a waiver of consent was granted because data were deidentified administrative records. This study is reported following the Strengthening the Reporting of Observational Studies in Epidemiology (STROBE) reporting guideline.

A serial cross-sectional analysis was performed for all unintentional fatal drug overdoses between January 1, 2009, and December 31, 2018. The burden of fatal drug overdose was calculated in years of life lost (YLL), computed by subtracting the age at death from the standard life expectancy for each decedent. Deaths were stratified by age (0-14, 15-19, 20-29, 30-39, 40-49, 50-59, or ≥60 years) and sex. Life expectancy by age and sex was determined from the Social Security Administration Period Life Table.^5^ This procedure was repeated for all causes of death for comparison with drug overdose. Finally, change in mean life span associated with overdose was calculated as


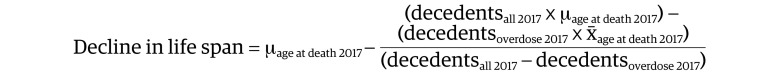
.

Analyses were performed using SPSS Statistics version 24 (IBM Corp). Data were analyzed on September 27, 2019.

## Results

There were 26 350 unintentional drug overdose deaths in Ohio from January 1, 2009, to December 31, 2018, and opioids were involved in 20 793 deaths (78.9%). Among 1 026 821 YLL, the groups who experienced highest YLL were white individuals (916 144 YLL [89.2%]), men (663 722 YLL [64.6%]), and individuals aged 30 to 39 years (328 007 YLL [31.9%]) or 20 to 29 years (259 144 YLL [25.2%]). Drug overdose was associated with 1 026 821 YLL and was the third leading cause of excess mortality after malignant neoplasms (3 944 244 YLL) and heart disease (3 235 989 YLL). The Figure illustrates drug overdose YLL among leading causes of excess mortality.

**Figure.**
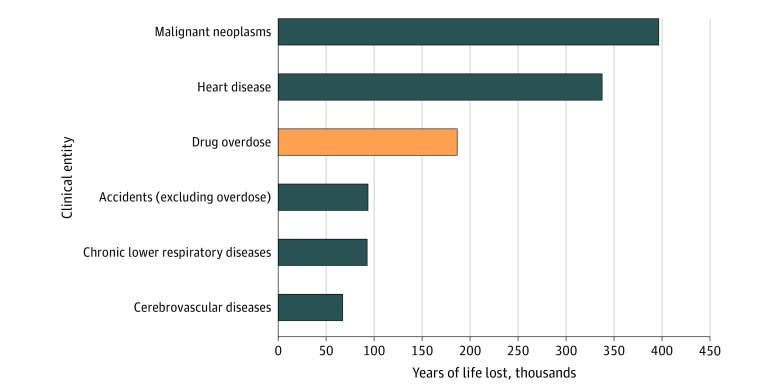
Top 6 Causes of Excess Mortality in Years of Life Lost in Ohio in 2017

All-cause mortality increased 14.2% during the period, with total annual YLL increasing from 1 607 512 YLL in 2009 to 1 836 220 YLL in 2018. A total of 38.2% of this increase was due to drug overdose. The next leading clinical entity, heart disease, accounted for just 12.7% of the increase.

The peak year for overdose deaths was 2017, during which Ohio experienced 187 006 overdose-related YLL, accounting 9.9% of all-cause excess mortality in Ohio and lowering the mean life span by 1.27 years. The Table presents mortality trends during the study.

**Table.** Annual Trends in All-Cause Mortality and the Top 3 Causes of Excess Mortality in Ohio From 2009 to 2018

Factor	2009	2010	2011	2012	2013	2014	2015	2016	2017	2018
**All deaths**										
Deaths, No.	107 061	108 458	110 705	112 049	112 915	114 343	118 014	119 574	123 650	123 596
Total YLL	1 607 512	1 600 450	1 628 744	1 646 358	1 662 433	1 686 541	1 756 972	1 821 864	1 893 923	1 836 220
YLL per death, mean (SD)	15.0 (13.0)	14.8 (12.9)	14.7 (12.9)	14.7 (12.8)	14.7 (12.8)	14.7 (12.7)	14.9 (12.9)	15.2 (13.2)	15.3 (13.2)	14.9 (12.8)
Age at death, mean (SD), y	73.1 (17.8)	73.6 (17.8)	73.6 (17.8)	73.7 (17.7)	73.6 (17.7)	73.5 (17.6)	73.4 (17.9)	72.9 (18.2)	72.8 (18.2)	73.4 (17.8)
**Malignant neoplasm**										
Deaths, No.	25 076	25 030	24 967	25 199	24 918	25 390	25 367	25 507	25 647	24 771
Total YLL	399 244	393 169	391 473	396 489	393 441	399 724	396 571	395 538	397 670	380 924
YLL per death, mean (SD), y	15.9 (9.7)	15.7 (9.6)	15.7 (9.5)	15.7 (9.6)	15.8 (9.5)	15.7 (9.4)	15.6 (9.4)	15.5 (9.3)	15.5 (92)	15.4 (9.1)
Age at death, mean (SD), y	70.7 (13.5)	71.1 (13.5)	71.1 (13.4)	71.0 (13.4)	70.9 (13.3)	71.0 (13.3)	71.1 (13.2)	71.3 (13.2)	71.3 (13.0)	71.4 (12.9)
**Heart disease**										
Deaths, No.	25 898	26 072	26 179	26 304	26 761	26 934	28 026	27 407	28 000	28 708
Total YLL	311 390	311 112	309 676	315 205	320 219	322 056	334 268	333 110	338 526	340 428
YLL per death, mean (SD)	12.0 (9.5)	11.9 (9.4)	11.8 (9.3)	12.0 (9.5)	12.0 (9.4)	12.0 (9.4)	11.9 (9.4)	12.2 (9.5)	12.1 (9.4)	11.9 (9.2)
Age at death, mean (SD), y	77.3 (14.6)	77.4 (14.6)	77.6 (14.5)	77.4 (14.6)	77.4 (14.7)	77.4 (14.6)	77.5 (14.6)	77.1 (14.8)	77.2 (14.6)	77.6 (14.5)
**All overdose deaths**										
Deaths, No.	1389	1511	1720	1864	2065	2476	2986	3939	4786	3614
Total YLL	53 720	58 574	65 693	72 117	78 737	96 595	117 738	155 632	187 006	141 008
YLL per death, mean (SD)	38.7 (10.9)	38.8 (11.2)	38.2 (11.1)	38.7 (10.8)	38.1 (11.1)	39.0 (11.1)	39.4 (11.1)	39.5 (11.2)	39.1 (11.3)	39.0 (11.4)
Age at death, mean (SD), y	41.8 (13.3)	41.7 (13.3)	42.4 (13.3)	41.7 (12.8)	42.3 (13.3)	41.3 (13.3)	40.8 (13.2)	40.7 (13.2)	41.2 (13.4)	41.3 (13.5)
**Opioid-involved overdose deaths**										
Deaths, No.	780	978	1130	1239	1511	1989	2565	3423	4112	3066
Total YLL	31 115	38 693	44 354	49 475	59 116	78 879	103 488	137 382	163 407	121 689
YLL per death, mean (SD)	39.9 (10.4)	39.6 (11.1)	39.3 (10.8)	39.9 (10.6)	39.1 (10.8)	39.7 (10.9)	40.3 (10.7)	40.1 (11.0)	39.7 (11.0)	39.7 (11.0)
Age at death, mean (SD), y	40.3 (12.5)	40.7 (13.3)	41.1 (12.7)	40.2 (12.4)	41.0 (12.8)	40.5 (12.9)	39.8 (12.6)	40.0 (12.8)	40.4 (12.9)	40.5 (12.9)
**Non–opioid-involved overdose deaths**										
Deaths, No.	609	533	590	625	554	487	421	516	674	548
Total YLL	22 605	19 881	21 339	22 642	19 621	17 716	14 249	18 250	23 599	19 320
YLL per death, mean (SD)	37.1 (11.3)	37.3 (11.1)	36.2 (11.2)	36.2 (10.9)	35.4 (11.5)	36.4 (11.6)	33.8 (11.7)	35.4 (11.7)	35.0 (12.2)	35.3 (12.8)
Age at death, mean (SD), y	43.8 (13.9)	43.5 (13.1)	45.0 (13.8)	44.7 (13.0)	45.6 (14.1)	44.7 (14.2)	47.5 (14.3)	45.6 (14.4)	46.0 (14.7)	46.0 (15.4)

Abbreviation: YLL, years of life lost.

## Discussion

During the course of a decade, Ohio lost more than 1 million years of human life to drug overdose. Drug overdose contributed more to an observed increase in all-cause mortality than any other cause and was associated with reduced mean life span in 2017. Drug overdose was the third leading cause of excess mortality in Ohio, a fact obfuscated by previous reports that included these deaths in the category of unintentional injury fatalities and reported incidence alone without accompanying YLL.^6^ Our analysis is limited by the use of death certificate data with possibly incomplete cause-of-death reporting.

We recommend that YLL be monitored in states experiencing increased drug overdose burden and that drug overdose YLL be compared with other clinical entities to better contextualize the current era of opioid misuse and overdose. Additionally, drug overdoses should be reported separately from other unintentional injuries in epidemiological research, as reporting them together obscures drug overdose as a leading cause of preventable mortality.
